# A Proxy Outcome Approach for Causal Effect in Observational Studies: A Simulation Study

**DOI:** 10.1155/2014/872435

**Published:** 2014-02-18

**Authors:** Wenbin Liang, Yuejen Zhao, Andy H. Lee

**Affiliations:** ^1^National Drug Research Institute, Health Science, Curtin University, G.P.O. Box U 1987, Perth, WA 6845, Australia; ^2^Northern Territory Department of Health, Darwin, NT 0800, Australia; ^3^School of Public Health, Health Science, Curtin University, Perth, WA 6845, Australia

## Abstract

*Background*. Known and unknown/unmeasured risk factors are the main sources of confounding effects in observational studies and can lead to false observations of elevated protective or hazardous effects. In this study, we investigate an alternative approach of analysis that is operated on field-specific knowledge rather than pure statistical assumptions. *Method*. The proposed approach introduces a proxy outcome into the estimation system. A proxy outcome possesses the following characteristics: (i) the exposure of interest is not a cause for the proxy outcome; (ii) causes of the proxy outcome and the study outcome are subsets of a collection of correlated variables. Based on these two conditions, the confounding-effect-driven association between the exposure and proxy outcome can then be measured and used as a proxy estimate for the effects of unknown/unmeasured confounders on the outcome of interest. Performance of this approach is tested by a simulation study, whereby 500 different scenarios are generated, with the causal factors of a proxy outcome and a study outcome being partly overlapped under low-to-moderate correlations. *Results*. The simulation results demonstrate that the conventional approach only led to a correct conclusion in 21% of the 500 scenarios, as compared to 72.2% for the alternative approach. *Conclusion*. The proposed method can be applied in observational studies in social science and health research that evaluates the health impact of behaviour and mental health problems.

## 1. Background

Due to lack of randomization, estimates obtained from observational studies are often affected by uncontrolled or unmeasured confounding effects. Several methods have been proposed to deal with the problem [1–11], but their application relies on assumptions about the distribution of the unknown confounding factor(s) in relation to the outcome, the exposure, and other known covariates.

The present study investigates an alternative approach of analysis that makes use of field-specific knowledge to determine a proxy outcome, which then will be employed to estimate uncontrolled confounding effects. A proxy outcome should satisfy the following conditions: (i) the exposure of interest is not a cause for the proxy outcome; and (ii) causes of the proxy outcome and the main outcome are subsets of a collection of correlated variables. If condition one is satisfied, then it is certain that the observed association between the exposure and the proxy outcome is completely driven by confounding effects. Nevertheless, condition one may be relaxed to some extent, for example, when it is certain that the confounding effect is by far stronger than possible causal effect. When condition two is satisfied, confounders for the proxy outcome and the outcome of interest are similar or at least correlated. For example, various forms of physical and mental health outcomes can be affected by a cluster of socioeconomic, behavioural, psychological, and genetic factors [12–22]. Researchers can apply their field knowledge and experience to determine the best proxy outcome for their outcome and exposure of interest.

Intuitively, let *y* and $\tilde{y}$ be the main outcome and the proxy outcome, respectively; *r* denotes the possible risk of *y* due to a given level of exposure; *t*_0_ and *t*_1_ the unexposed and exposed person-time at risk; *d*_0_ and *d*_1_ represent the number of cases of *y* observed in *t*_0_ and *t*_1_, whereas *d*_0_′ and *d*_1_′ are the number of cases of $\tilde{y}$ observed in *t*_0_ and *t*_1_, respectively. Further, suppose that *P*(*d*_0_) and *P*(*d*_1_) define the expected probability of one or more sufficient causes that the exposure of interest is not involved, occurring for *y* in a unit of person-time at risk within *t*_0_ and *t*_1_, respectively; correspondingly, *P*(*d*_0_′) and *P*(*d*_1_′) define the expected probability of one or more sufficient causes occurring for $\tilde{y}$ in a unit of person-time at risk within *t*_0_ and *t*_1_, respectively. Then, the observed crude risk ratios are

(1) $$\begin{array}{c} \frac{d_{1}/t_{1}}{d_{0}/t_{0}}=\frac{\left\{r\left[1-P\left(d_{1}\right)\right]+P\left(d_{1}\right)\right\}}{P\left(d_{0}\right)}\\ \frac{d_{1}^{\prime }/t_{1}}{d_{0}^{\prime }/t_{0}}=\frac{P\left(d_{1}^{\prime }\right)}{P\left(d_{0}^{\prime }\right)}. \end{array}$$

If the sufficient causes of *y* and $\tilde{y}$ are the same, largely overlapped, or strongly correlated, then

(2) $$\begin{array}{c} \frac{P\left(d_{1}\right)}{P\left(d_{0}\right)}=\frac{P\left(d_{1}^{\prime }\right)}{P\left(d_{0}^{\prime }\right)},\\ \frac{d_{1}/t_{1}}{d_{0}/t_{0}}=\frac{d_{1}^{\prime }/t_{1}}{d_{0}^{\prime }/t_{0}} \end{array}$$

will be observed if the exposure is not causal for *y* (*r* = 0), whereas

(3) $$\begin{array}{c} \frac{d_{1}/t_{1}}{d_{0}/t_{0}}>\frac{d_{1}^{\prime }/t_{1}}{d_{0}^{\prime }/t_{0}} \end{array}$$

will be observed if the exposure is causal for *y* (*r* > 0). Given this strict assumption, the method has been successfully applied in time series analysis recently, whereby the proxy outcome was described as a control series [23, 24]. For example, the study by Herttua and colleagues investigated the effect of alcohol price (exposure) on alcohol-related mortality (outcome of interest), while coronary operations were used as the control series (proxy outcome) [23]. Nevertheless, such a method may remain valid when assumptions are relaxed and can be applied to different study designs. Because the details of underlying causal mechanisms for an outcome (i.e., all sufficient causes) are typically unknown, it is best to ascertain the validity of the method through simulations. Therefore, we conduct a simulation study to test its application, focusing on situations when the causes of *y* and $\tilde{y}$ are only partly overlapped and have only low to moderate strength of association.

## 2. Method

### 2.1. Simulation Design

The simulation process follows the sufficient cause model [25]. In the simulation for an event to occur, at least one sufficient cause has to occur, which comprises the occurrence of two matched causal components and absence of any competing event. In addition, a randomly distributed small error term is introduced to ensure that perfect prediction (which interrupts the computing process) will not occur. To account for the fact that only certain real causal factors are known yet some of the noncausal factors are mistaken as causal factors, a collection of variables are included to encompass exposure, causal factors, and noncausal factors, while a subset from the pool provides the known variables. All simulations are performed within the STATA package release 12.

The simulations contain 500 replicates, with each replicate being generated through an independent process. There are seven simulation steps involved in each replicate.

1. Generate a collection of correlated binary variables, **X**_40×50000_ = {*X*_*in*_}, *i* = (1, 2, 3,…, 40), and *n* = (1, 2, 3,…, 50000). For each *n*, *X*_*in*_ is set to 1 if an intermediate process variable *T*_*in*_ ≥ 0.75 and 0 otherwise, where *T*_*in*_ = *V*_*in*_ *P*_*i*_ + *U*_*n*_(1 − *P*_*i*_) is a combination of a variable component (*V*_*i*_) and an unique component (*U*) for each *i*, both being uniform [0,1) distributed random variables, and *P*_*i*_ is a random proportion drawn from a uniform [0.3, 0.8) distribution. The range [0.3, 0.8) is chosen in order to set a low to moderate level of correlation among *X*. The mean (standard deviation), 25th, 50th, and 75th percentiles of the correlation coefficients for the matrix **X** are 0.29 (0.12), 0.20, 0.26, and 0.34, respectively.
2. Determine the real causes for outcomes of interest *A*, *B*, and proxy outcome *C*. Real causes for *A*, *B*, and *C* are subsets of **X** in which *X*_*i*_ and *X*_*i*+10_, *i* = (1,2, 3,…, 10) form ten matched pairs. Let *F*_*j*,*i*_ indicate the factual causes, *j* = (*A*, *B*, *C*) and *i* = (1,2, 3,…, 10). In this simulation, we force *F*_*A*,1_ = 1 and *F*_*B*,1_ = *F*_*C*,1_ = 0; that is, *X*_1_ is causal for *A*, but not causal for *B* and *C*. For *i* = (2, 3,…, 10), *F*_*j*,*i*_ is a random value drawn from the Bernoulli distribution with probability of success = 0.5, value of success = 1, and value of failure = 0. For example if *F*_*A*,2_ = 1, then the pair of *X*_2_ and *X*_12_ is a cause of *A*.
3. Generate competing events for outcomes *A*, *B*, and *C*. Let *E*_*j*,*n*_ denote the competing events for outcomes *A*, *B*, and *C*, *j* = (*A*, *B*, *C*) and *n* = (1, 2, 3,…, 50000). *E* is a Bernoulli distributed random variable with a probability of success = 0.1, value of success = 1, and value of failure = 0. *E* is also independent of *X*.
4. Generate background errors for outcomes *A*, *B*, and *C*. Let *Q*_*j*,*n*_ denote “background” sufficient causes, *j* = (*A*, *B*, *C*) and *n* = (1, 2, 3,…, 50000). *Q* is a Bernoulli distributed random variable with a probability of success = 0.05, value of success = 1, and value of failure = 0. *Q* is independent of *E* and *X*. *Q* services as small random error, and it is introduced to smooth the computing process only.
5. Determine the status (occur or not occur) of outcomes *A*, *B*, and *C*. Let *Y*_*j*,*n*_ where *j* = (*A*, *B*, *C*) and *n* = (1, 2, 3,…, 50000)  denote the status of outcomes *A*, *B*, and *C*. Value of each *Y*_*j*,*n*_ is determined as follows. For each observation *n*, *Y*_*j*,*n*_ = 1 (outcome occurred) if *Q*_*j*,*n*_ = 1, or for *i* = (1, 2, 3,…, 10), ∑_*i*_ *X*_*in*_ *X*_(*i*+10)*n*_ *F*_*j*,*i*_ ≥ 1 when *E*_*j*,*n*_ = 0; Otherwise *Y*_*j*,*n*_ = 0 (outcome not occurred).
6. Determine the known/suspected (not necessary the fact) “causal” factors (except *X*_1_) for outcomes *A* and *B*. The known/suspected “causal” factors for outcomes *A*, *B*, and *C* are determined for *X*_*i*_ for *i* = (1,2, 3,…, 40). Let *K*_*j*,*i*_ denote the researcher's knowledge (not necessary the fact) of causes for outcomes *A* and *B*. Let *j* = (*A*, *B*), and *K*_*j*,*i*_ indicates a known “causal” factor. Because *X*_1_ is the exposure of interest, so we force each *K*_*j*,*i*_ = 1 when *i* = 1. For *i* = (2, 3,…, 20), *K*_*j*,*i*_ is a random value drawn from a Bernoulli distribution with a probability of success = 0.5. For *i* = (21, 22, 23,…, 40), *K*_*j*,*i*_ is a random value drawn from the Bernoulli distribution with a probability of success = 0.15, value of success = 1, and value of failure = 0. The difference in the success rates between the two groups indicates that a real causal factor is more likely to be acknowledged than a noncausal factor.
7. Estimate the effects of *X*_1_ on outcomes *A* and *B* based on the fact model, and compare the conventional approach with the proposed approach. Let *G*_*j*,*n*_, *j* = (*A*, *B*), *n* = (1, 2, 3,…, 50000), be the presence of causes (except *X*_1_ and *X*_11_) for outcomes *A* and *B* for each observation. For each *n*, *G*_*j*,*n*_ = 1 for *i* = (2, 3 …, 10), if ∑_*i*_ *X*_*in*_ *X*_(*i*+10)*n*_ *F*_*j*,*i*_ ≥ 1; *G*_*j*,*n*_ = 0 otherwise.

The true effects of *X*_1_ on outcomes *A* and *B* based on the fact model are estimated by

(4) P(Yj,n=1 ∣ X1n,Gj,n)  =exp⁡(βj,1X1n+βjGj,n)1+exp⁡(βj,1X1n+βjGj,n),

where *β*_*j*,*i*_ is the estimated real effect of *X*_*i*_ on outcome *j* for *j* = (*A*, *B*). To estimate the effects of *X*_1_ on outcomes *A* and *B* based on known/suspected confounders and applying standard multivariate logistic regression as the adjustment method, we have

(5) P(Yj,n=1 ∣ Xin,Kj,i)  =exp⁡(βj,1′X1n+∑i=240βj,i′XinKj,i)1+exp⁡(βj,1′X1n+∑i=240βj,i′XinKj,i),

where *β*_*j*,*i*_′ is the estimated effect of *X*_*i*_ on outcome *j* for *j* = (*A*, *B*). To estimate the confounding effects on *X*_1_ on outcomes *A* and *B* using the proxy outcome *C*, the logistic model for adjustment becomes

(6) P(YC,n=1 ∣ Xin,Kj,i)  =exp⁡⁡(β~j,1′X1n+∑i=240β~j,i′XinKj,i)1+exp⁡⁡(β~j,1′X1n+∑i=240β~j,i′XinKj,i),

where ${\tilde{\beta }}_{j,i}^{\prime }$ is the estimated effect of *X*_*i*_ on outcome *C* (proxy outcome) for *j* = (*A*, *B*).

The adjusted effects of *X*_1_ on *A* and *B* are ${\hat{\beta }}_{A,1}=\beta_{A,1}^{\prime }-{\tilde{\beta }}_{A,1}^{\prime }$ and ${\hat{\beta }}_{B,1}=\beta_{B,1}^{\prime }-{\tilde{\beta }}_{B,1}^{\prime }$, respectively.

### 2.2. Classification of Effect of *X*_1_ on *A* and *B*

Based on the fact model, *X*_1_ increases risk of outcome *A* if *β*_*A*,1_ > 0.05 and *P* value for *β*_*A*,1_ < 0.05; otherwise *X*_1_ has no effect on *A*. Also, *X*_1_ increases risk of outcome *B* if *β*_*B*,1_ > 0.05 and *P* value for *β*_*B*,1_ < 0.05; otherwise *X*_1_ has no effect on *B*. The same effect patterns hold analogously for the conventional model and the alternative approach by replacing the regression coefficient *β*_*j*,1_ with *β*_*j*,1_′ and ${\hat{\beta }}_{j,1}$, respectively. Classifications of the effect of *X*_1_ based on the fact model are then used as the gold standard to compare with the classifications based on the conventional approach and the alternative approach.

### 2.3. Empirical Application

A simple example is provided to clarify the methodology. For additional illustration of the proxy outcome method to adjust for residual confounding effects, interested readers are referred to the first author's recently published research [26]. Briefly, when investigating the effect of alcohol use (exposure) on general health status (outcome), both measured and unmeasured confounding factors are involved. Many of these confounding factors are clustered within the family such as socioeconomic determinants, environmental factors, lifestyle, and genetic susceptibility. Although current alcohol use by adults does not produce any physiological effect on their children's current health, observed effect of current alcohol use (exposure) on their children's health status (proxy outcome) can be used as an approximation of confounding effects.

This example used the data from the 2010 National Health Interview Survey. A first logistic regression model was fitted to compare the likelihood of having undesirable (poor or fair) health status (outcome) between lifetime abstainers and current light drinkers (exposure). A second logistic regression model was then applied to compare the likelihood of having undesirable health status in the children (proxy outcome) in relation to the drinking status of their parents. To adjust for confounding effects, natural logarithm of the odds ratios from the second model was introduced as an offset variable into the first model.

## 3. Results

Estimates based on the knowledge and conventional model from one replicate are shown in Tables 1 and 2 as an example. Both outcomes *A* and *B* are treated as the outcomes of interest, while outcome *C* is used as the proxy outcome. In this replicate, between outcomes *A* and *C*, there are four common causal factors. These account for 33% and 40% of all causal factors for *A* and *C*, respectively. Except for the exposure of interest (*X*_1_), 54% of causal factors for *A* are known. The true effect of *X*_1_ on outcome *A* (*β*_*A*,1_) based on the fact model is 1.27 (*P* value < 0.001) indicating that *X*_1_ is a real causal factor to *A*. The estimated effect based on the conventional approach (*β*_*A*,1_′) is 0.79 (*P* value < 0.001). The estimated effect based on the alternative approach (${\hat{\beta }}_{A,1}$) is 0.79 − 0.09 = 0.70. Both the conventional approach and alternative approach lead to the same correct conclusion that *X*_1_ is a causal factor to *A*.

Between outcomes *B* and *C* there are also four common factors. These account for 67% and 40% of all causal factors for *B* and *C*, respectively. Except the exposure of interest (*X*_1_), 33% of causal factors for *B* are known. The true effect of *X*_1_ on outcome *B* (*β*_*B*,1_) based on the fact model is −0.024 (*P*  value = 0.668) indicating that *X*_1_ is not a causal factor to *B*. The estimated effect based on the conventional approach (*β*_*B*,1_′) is 0.12 (*P*  value < 0.001). The estimated effect based on the alternative approach (${\hat{\beta }}_{B,1}$) is 0.12 − 0.16 = −0.04. Therefore, based on estimation from the conventional approach one would mistakenly draw the conclusion that “*X*_1_ is a causal factor to *B*.” However, given that ${\hat{\beta }}_{B,1}<0.05$, the alternative approach has led to the correct conclusion that *X*_1_ is not a causal factor to *B*.

Table 3 summarises findings from the 500 replicates. Based on the fact model, the exposure of interest (*X*_1_) is classified as a causal factor for outcome *A* in all replicates. This is in perfect agreement with the simulation process that *X*_1_ is set to be a causal factor for *A*. In the simulation process, *X*_1_ is set to be a noncausal factor for *B*; in 97.4% of the 500 replicates, the fact model concludes that *X*_1_ is not a causal factor for *B*, but in 2.6% of the replicates the fact model concludes that *X*_1_ is a causal factor for *B*. The disagreement between the simulation process and the fact model in these 2.6% replicates is a result of type I error (setting the two-sided confidence interval to 95%). Nevertheless, in all replicates, both the conventional and alternative approaches have classified *X*_1_ as a causal factor for *A*. When comparing the estimates between the conventional approach and the fact model, the two models have led to the same conclusion in only 21% of the replicates, while in 72.2% of the replicates, the alternative approach has led to the same conclusion as the fact model. When the simulation process is used as the gold standard for classification (i.e., *X*_1_ is causal for *A*, but not causal for *B*), the sensitivity of the new approach is 100%, and the specificity is 70.2%.

Table 4 presents results of the example. Alcohol use by adults has similar effects on their health status and their children's health status, whereas the effect on children's health status is mediated by confounding factors. To account for the uncontrolled confounding effects when estimating the effect of adults' alcohol use on their health status, an offset variable, which takes on the value of the natural logarithm of 1 for lifetime abstainers and 0.60 for current light drinkers, is added to the model. The adjustment changes the odds ratio from 0.54 (*P* < 0.001) to 0.90 (*P* = 0.38).

## 4. Discussion

In this study, we introduce a new analysis approach for causal effect. Although the new approach is only applicable to measurements of relative effects (i.e., risk ratios, odds ratios), it does not require any distributional assumption for the confounding variables in relation to the outcome, the exposure, and other known confounding variables. Instead, the approach merely assumes that the causes for the outcome of interest and proxy outcome are partly overlapped and correlated. The choice of an optimal proxy outcome is achievable by directly applying field expertise without advanced knowledge in statistics. The simulation results show that the alternative approach is far more accurate than the conventional approach in classifying causal associations, even under conditions of low to moderate correlation between the causes for the outcome and causes for the proxy outcome. The proposed approach appears to be suitable for observational studies in social science and health research that evaluate the health impact of behaviour and mental health problems, especially where clusters of causes for various outcomes are strongly correlated and overlapped in these fields [12, 27, 28].

It should be remarked that the analysis can only be performed when effects are measured by relative risk difference such as risk ratio or odds ratio. Another limitation is that false classification remains possible, even though the proposed method appears to have an advantage over the conventional approach. In this study, we demonstrate a new simulation process that incorporates the component causes, competing events, difference between the fact and the knowledge, to model realistic scenarios in observational studies. This simulation process could be further developed and used to determine how knowledge that deviates from the fact can introduce bias in estimates.

## 5. Conclusion

In conclusion, the proposed proxy outcome approach can be applied in observational studies in social science and health research that evaluate the health impact of behaviour and mental health problems.

## Figures and Tables

**Table 1:** Data example of a replicate/scenario, estimated effects (coefficients from logistic models) of exposure (*X*_1_), and known “causal”/confounding factors of *A* on *A* and proxy outcome *C*.

	Effects on *A *		Effects on *C *		Indicators for real causal factors (1 = Yes, 0 = No)	
	Coefficient	*P* value	Coefficient	*P* value	Causal to *A *	Causal to *C *
*X*_1_	0.79	0.000	0.09	0.011	1	0
*X*_2_	0.19	0.000	0.92	0.000	0	1
*X*_3_	0.69	0.000	0.03	0.360	1	0
*X*_4_	0.46	0.000	0.50	0.000	0	0
*X*_5_	∗		∗		1	1
*X*_6_	∗		∗		1	1
*X*_7_	0.87	0.000	0.29	0.000	1	0
*X*_8_	0.20	0.000	0.84	0.000	0	1
*X*_9_	0.04	0.293	0.80	0.000	0	1
*X*_10_	0.77	0.000	0.13	0.000	1	0
*X*_11_	∗		∗		1	0
*X*_12_	0.15	0.000	0.79	0.000	0	1
*X*_13_	∗		∗		1	0
*X*_14_	∗		∗		0	0
*X*_15_	0.85	0.000	0.91	0.000	1	1
*X*_16_	∗		∗		1	1
*X*_17_	0.73	0.000	0.10	0.009	1	0
*X*_18_	0.15	0.000	0.89	0.000	0	1
*X*_19_	∗		∗		0	1
*X*_20_	0.95	0.000	0.35	0.000	1	0
*X*_21_	∗		∗		0	0
*X*_22_	0.27	0.000	0.16	0.000	0	0
*X*_23_	0.25	0.000	0.19	0.000	0	0
*X*_24_	0.20	0.000	0.18	0.000	0	0
*X*_25_	∗		∗		0	0
*X*_26_	∗		∗		0	0
*X*_27_	∗		∗		0	0
*X*_28_	∗		∗		0	0
*X*_29_	0.50	0.000	0.47	0.000	0	0
*X*_30_	∗		∗		0	0
*X*_31_	∗		∗		0	0
*X*_32_	∗		∗		0	0
*X*_33_	∗		∗		0	0
*X*_34_	∗		∗		0	0
*X*_35_	∗		∗		0	0
*X*_36_	∗		∗		0	0
*X*_37_	∗		∗		0	0
*X*_38_	0.13	0.001	0.17	0.000	0	0
*X*_39_	∗		∗		0	0
*X*_40_	0.16	0.000	0.17	0.000	0	0

*indicates variable is not known as a “causal” factor for *A*, therefore is not included in the models.

**Table 2:** Data example of a replicate/scenario, estimated effects (coefficients from logistic models) of exposure (*X*_1_), and known “causal”/confounding factors of *B* on *B* and proxy outcome *C*.

	Effects on *B *		Effects on *C *		Indicators for real causal factors (1 = Yes, 0 = No)	
	Coefficient	*P* value	Coefficient	*P* value	Causal to *B *	Causal to *C *
*X*_1_	0.12	0.001	0.16	0.000	0	0
*X*_2_	∗		∗		1	1
*X*_3_	∗		∗		1	0
*X*_4_	0.39	0.000	0.62	0.000	0	0
*X*_5_	∗		∗		0	1
*X*_6_	∗		∗		0	1
*X*_7_	0.24	0.000	0.43	0.000	0	0
*X*_8_	∗		∗		1	1
*X*_9_	∗		∗		0	1
*X*_10_	∗		∗		0	0
*X*_11_	∗		∗		0	0
*X*_12_	1.22	0.000	0.88	0.000	1	1
*X*_13_	1.15	0.000	0.17	0.000	1	0
*X*_14_	0.23	0.000	0.27	0.000	0	0
*X*_15_	0.16	0.000	1.04	0.000	0	1
*X*_16_	0.31	0.000	1.56	0.000	0	1
*X*_17_	0.13	0.000	0.19	0.000	0	0
*X*_18_	∗		∗		1	1
*X*_19_	∗		∗		0	1
*X*_20_	0.28	0.000	0.43	0.000	0	0
*X*_21_	∗		∗		0	0
*X*_22_	0.17	0.000	0.24	0.000	0	0
*X*_23_	∗		∗		0	0
*X*_24_	∗		∗		0	0
*X*_25_	∗		∗		0	0
*X*_26_	∗		∗		0	0
*X*_27_	∗		∗		0	0
*X*_28_	0.23	0.000	0.26	0.000	0	0
*X*_29_	0.31	0.000	0.58	0.000	0	0
*X*_30_	∗		∗		0	0
*X*_31_	∗		∗		0	0
*X*_32_	∗		∗		0	0
*X*_33_	∗		∗		0	0
*X*_34_	∗		∗		0	0
*X*_35_	∗		∗		0	0
*X*_36_	∗		∗		0	0
*X*_37_	∗		∗		0	0
*X*_38_	∗		∗		0	0
*X*_39_	∗		∗		0	0
*X*_40_	∗		∗		0	0

*indicates variable is not known as a “causal” factor for *B*, therefore is not included in the models.

**Table 3:** Effect of *X*_1_ on *A* and *B* based on the fact model, conventional approach, and the alternative approach.

Fact model	Conventional approach		Alternative approach (%)	
	*X*_1_ causal to *A* but not *B* (%)	*X*_1_ causal to *A *and *B* (%)	*X*_1_ causal to *A* but not *B* (%)	*X*_1_ causal to *A* and *B *(%)
*X*_1_ causal to *A* but not *B *	93 (18.6%)	394 (78.8%)	347 (69.4%)	140 (28.0%)
*X*_1_ causal to *A* and *B *	1 (0.2%)	12 (2.4%)	4 (0.8%)	9 (1.8%)

**Table 4:** Empirical application, predicting adults' health status (outcome of interest) and their children's health status (proxy outcome) by alcohol use (proxy outcome).

	Have undesirable health status in adults			Have undesirable health status in children		
	Odds Ratio	95% confidence interval		Odds ratio	95% confidence interval	
Parents' drinking behaviour						
Lifetime abstainers	1.00	Reference		1.00	Reference	
Current light drinkers	0.54	0.42	0.69	0.60	0.50	0.71
